# Transcatheter Arterial Embolization (TAE) of Uterine Artery with Gelatin Sponge for Cesarean Scar Pregnancy: A Current State of the Art Review

**DOI:** 10.3390/gels12010044

**Published:** 2026-01-01

**Authors:** Roberto Minici, Francesco Tiralongo, Massimo Venturini, Federico Fontana, Filippo Piacentino, Melania Nicoletta, Andrea Coppola, Giuseppe Guzzardi, Francesco Giurazza, Fabio Corvino, Domenico Laganà

**Affiliations:** 1 Department of Clinical and Experimental Medicine, Magna Graecia University, 88100 Catanzaro, Italy; 2 Radiology Unit, Dulbecco University Hospital, 88100 Catanzaro, Italy; melania.nicoletta@gmail.com; 3 Radiology Unit 1, Department of Medical Surgical Sciences and Advanced Technologies “GF Ingrassia”, University Hospital Policlinico “G. Rodolico-San Marco”, University of Catania, 95123 Catania, Italy; tiralongofrancesco91@hotmail.it; 4 Diagnostic and Interventional Radiology Unit, ASST Settelaghi, Insubria University, 21100 Varese, Italy; massimo.venturini@uninsubria.it (M.V.); federico.fontana@uninsubria.it (F.F.); filippo.piacentino@asst-settelaghi.it (F.P.); andrea.coppola@asst-settelaghi.it (A.C.); 5 Imagerie Vasculaire et Interventionnelle, Centre Hospitalier Princesse Grace, 98000 Monaco, Monaco; giuguzzardi@gmail.com; 6 Interventional Radiology Department, AORN “A. Cardarelli”, 80131 Naples, Italy; francescogiurazza@hotmail.it (F.G.); fabio.corvino@unipa.it (F.C.)

**Keywords:** cesarean scar pregnancy, uterine artery embolization, transcatheter arterial embolization, gelatin sponge, gelfoam, placenta accreta spectrum, fertility preservation, obstetric hemorrhage, methotrexate, interventional radiology

## Abstract

Cesarean scar pregnancy (CSP) carries a high risk of severe hemorrhage and potential loss of fertility. This narrative review summarizes current evidence on uterine artery embolization (UAE) using absorbable gelatin sponge (GS), focusing on GS preparation, procedural approaches, and reported outcomes. PubMed/MEDLINE, Scopus, and Google Scholar were searched from January 2015 to 31 December 2024 for peer-reviewed studies reporting UAE with GS for CSP (GS alone or combined with intra-arterial methotrexate and/or adjunct particles). Fifty studies (N = 3139) were included. Technical success was 3133/3139 (~99.8%) and clinical success was 2975/3139 (~94.8%), with most cohorts reporting high clinical control. Severe complications were infrequently reported (typically ~2–4% in most series). Menstrual function, when assessed, generally recovered within ~1–2 months. Subsequent pregnancy outcomes were inconsistently reported and follow-up durations were heterogeneous, predominantly in retrospective designs. Overall, UAE with GS appears effective for hemostasis in CSP and may reduce escalation to hysterectomy in appropriately selected patients. Standardized reporting of GS preparation and outcomes, as well as prospective multicenter registries/studies, are needed to refine best practices.

## 1. Introduction

Cesarean scar pregnancy (CSP) is a condition in which an early embryo implants within the cesarean scar defect (CSD), an area of myometrial disruption resulting from a previous cesarean section [1,2]. The prevalence of CSP has been rising, likely due to an increasing rate of primary cesarean deliveries and a declining trend in vaginal births after cesarean. Currently, CSP accounts for approximately 6% of all ectopic pregnancies [3]. Growing evidence suggests that CSP may represent an early stage in the spectrum of placenta accreta disorders [4]. Trophoblastic invasion occurs at the site of a uterine scar, which is structurally weakened by fibrotic remodeling, predisposing the uterus to complications such as rupture, severe hemorrhage, and other adverse outcomes [5].

There is widespread agreement that CSP should be promptly terminated upon diagnosis to prevent life-threatening complications [6]. Contemporary interventional radiology recommendations in obstetric hemorrhage and abnormal placentation recognize transcatheter techniques, including uterine artery embolization, as uterus-sparing options when expertise and resources are available [7]. Bilateral uterine artery embolization (UAE)—not to be confused with uterine fibroid embolization (UFE), which selectively targets benign tumors and their feeder arteries—is specifically aimed at reducing vascular perfusion in the pathological gestational site, serving as a hemostatic intervention in pregnancy-related pathology [8]. UAE has emerged as a key treatment strategy, either as a standalone intervention or in combination with other medical and surgical approaches, such as methotrexate (MTX) administration or uterine curettage [9,10,11,12]. The primary objective of UAE is to manage CSP effectively while minimizing the risk of massive hemorrhage and hysterectomy, thereby preserving uterine integrity and maintaining the potential for future pregnancies [13,14].

Although many different embolic agents are commonly used in transcatheter embolizations [15,16,17,18,19,20,21,22,23,24], UAE most often employs particles/microspheres, such as polyvinyl alcohol (PVA) particles, and gelatin sponge (GS) [25]. UAE using particles such as PVA is widely performed in Western countries due to its predictable efficacy and favorable safety profile [26]. However, gelatin sponge (GS) embolization retains distinct characteristics that warrant consideration and may offer advantages in specific scenarios [27]. Firstly, GS is significantly more cost-effective than PVA [28]. In Europe, a single package of GS costs approximately EUR 10–60, whereas a vial of PVA ranges from EUR 150 to 350. This cost disparity can be particularly relevant in resource-limited settings and in healthcare systems where the financial burden of the procedure falls directly on the patient or private insurance [29]. Secondly, GS embolization provides temporary occlusion [30], whereas PVA leads to permanent vessel occlusion [31]. Compared with calibrated PVA particles/microspheres (with predefined size distributions and standardized delivery characteristics), GS is resorbable and inexpensive. However, it is intrinsically more operator-dependent because the preparation method and resulting particle size distribution can vary widely. This variability may influence distal penetration and the risk of non-target embolization [32]. However, it remains unclear whether these differences affect the likelihood of achieving a future successful pregnancy in women who have not yet fulfilled their reproductive plans [33].

Hence, UAE with GS is an established clinical practice for the management of CSP worldwide [14,34,35]. The current body of scientific literature on this topic is largely composed of retrospective case series and case reports, with most data originating from Eastern countries, particularly China [36]. While endovascular embolization is a widely validated clinical procedure, the use of GS for UAE is hampered by substantial variability in its preparation techniques, as authors employ diverse methodologies [30]. This lack of standardization, combined with the low granularity of published studies, contributes to significant heterogeneity in the available data. Consequently, less experienced interventionalists who are newly adopting this technique may encounter uncertainty when interpreting the literature and applying it to clinical practice. Therefore, this review aims to provide a comprehensive overview of UAE with GS as a first-line therapy for CSP, synthesizing current evidence on preparation methods, procedural techniques, and clinical outcomes, and identifying knowledge gaps critical for standardizing practice and preserving fertility.

## 2. Data Collection Strategy

A comprehensive literature search was conducted using online databases (i.e., PubMed/Medline, Google Scholar, and Scopus) to identify peer-reviewed studies examining the outcomes of UAE with GS in patients suffering from CSP. The search covered articles published in the last ten years (from January 2015 to December 2024). The last search was performed on 31 December 2024. The PubMed/MEDLINE search string was: (“cesarean scar pregnancy” OR “caesarean scar pregnancy” OR “scar pregnancy” OR “ectopic pregnancy” OR “extrauterine pregnancy”) AND (“uterine artery embolization” OR “uterine artery embolisation” OR “transcatheter arterial embolization” OR “transcatheter arterial embolisation” OR “TAE” OR “UAE” OR “embo*”). Equivalent syntax was adapted for Scopus and Google Scholar. The complete search syntaxes (including database-specific field tags/limits and any applied filters) are provided in Supplementary File S2 for transparency and reproducibility. Titles and abstracts were screened to identify studies relevant to the UAE with GS as a first-line therapy in patients suffering from CSP, while unrelated research was excluded. Keywords such as “gelatin sponge” or “gelfoam” or “absorbable gelatin sponge” were searched to identify the embolic agent used and, where necessary, the full text was also screened. The analysis encompasses cases where UAE was performed as a standalone intervention to induce gestational sac resolution, as well as those where it was employed as a preparatory step before systemic intravenous methotrexate administration and/or uterine-sparing procedures such as dilation and curettage (D&C). Studies on transcatheter arterial chemoembolization (i.e., drugs, such as MTX, were administered via an intra-arterial transcatheter route during UAE) and studies where it was unclear whether reported complications were solely attributable to UAE or to subsequent pharmacologic or surgical interventions were also included. Studies combining UAE with intra-arterial MTX were included and analyzed as a predefined subgroup. The overarching objective in all these scenarios was to effectively treat CSP while avoiding major surgical interventions, particularly hysterectomy. The following exclusion criteria were applied: (I) studies lacking sufficient details on treatment methods or outcomes; (II) UAE was not the primary treatment for CSP but was instead performed as a salvage therapy for massive hemorrhage following mechanical interventions, such as curettage, or after pharmacologic induction of pregnancy termination; (III) studies were not considered if outcome assessments focused on subsequent pharmacologic or surgical treatments rather than UAE itself; (IV) non-English publications; (V) in vitro or animal studies; (VI) case series with fewer than 5 patients, case reports, or letters to the editor. Case reports were excluded to reduce publication bias toward unusual outcomes and to focus on studies with more stable, comparable estimates of efficacy and safety. After duplicates were removed using EndNote, titles and abstracts were screened to identify eligible studies, followed by a full-text review to confirm inclusion. Additionally, the reference lists of selected studies were manually examined for any further relevant literature. Two independent researchers conducted the screening process, resolving any discrepancies through mutual agreement. As per institutional protocol, a third researcher was designated to mediate disagreements, though his intervention was ultimately unnecessary. The PRISMA flow diagram is provided in Supplementary File S1. We additionally performed a design-informed study quality/risk-of-bias appraisal (adapted Newcastle–Ottawa Scale framework), reported as table in Supplementary File S3. The review protocol was not prospectively registered.

Table 1 provides an overview of the studies included in the review, summarizing their findings on UAE with GS for the management of CSP. Additionally, the GS preparation method and outcomes are reported, along with the specific effect of UAE on the menstrual cycle when specified in the original studies. Categorical variables are presented as percentages, whereas continuous variables are reported as either mean (standard deviation) or median (interquartile range: Q1–Q3), depending on the data distribution. The included studies were further divided into three subgroups according to the use of GS as the sole embolic agent, GS combined with intra-arterial administration of methotrexate, and GS plus particles such as PVA.

Technical success is defined as the ability to achieve blood stasis in uterine arteries at the end of the embolization procedure. Clinical success was defined as successful completion of conservative management without unplanned escalation (e.g., uncontrolled bleeding requiring emergency hysterectomy/major surgery or other non-planned rescue treatment) within the assessment window reported by each study (index hospitalization and/or follow-up). Because clinical success definitions and follow-up varied across cohorts, outcomes were extracted as reported and interpreted descriptively. The safety outcomes analyzed in the included studies specifically refer to UAE and do not account for complications related to subsequent pharmacologic or surgical treatments (e.g., methotrexate administration and uterine curettage). If the cause of the complications was not specified in the study, the complications were still reported in the review. When not otherwise specified in individual studies, complications were graded according to the SIR classification. In brief, the SIR system classifies adverse events as minor (I: no therapy/nominal therapy) versus major (II–V: requires therapy and/or hospitalization, may result in permanent sequelae, with death as the most severe category) [37].

**Table 1:** Summary of the 50 studies included in the literature review (January 2015–31 December 2024) reporting UAE with gelatin sponge for CSP.

Reference	Country	Study Design	Sample Size (N)	Age (Years)	Gestational Age (Days)	Embolic Agent	GS Form/Size	MTX Dose/Route	Technical Success Rate, n/N (%)	Clinical Success Rate, n/N (%)	Severe Complication Rate, n/N (%)	Reduced Menstrual Blood Volume, n/N (%)	Menstrual Recovery (Months)
Pecorino, 2024 [38]	Italy	Retrospective cohort study	10	34 (5.10)	57.26 (11.83)	GS	particles	-	10/10 (100%)	10/10 (100%)	0/10 (0%)	NR	NR
Ma, 2024 [39]	China	Retrospective cohort study	10	NR	NR	GS	particles 0.9–1.2 mm	-	10/10 (100%)	8/10 (80%)	0/10 (0%)	NR	NR
Rui, 2024 [40]	China	Retrospective cohort study	39	32.8 (3.80)	46.9 (9.70)	GS	particles 1 mm	-	39/39 (100%)	39/39 (100%)	0/39 (0%)	9/39 (22.20%)	NR
Gao, 2023 [41]	China	Retrospective cohort study	66	34.71 (5.91)	51.57 (8.85)	GS	particles 0.56–0.71 mm and 0.71–1 mm	-	66/66 (100%)	64/66 (96.97%)	2/66 (3.03%) Severe vaginal bleeding (n = 2)	NR	1.26 (0.24)
Wang, 2023 [42]	China	Retrospective cohort study	118	31.12 (5.39)	50.50(42–60)	GS	particles 0.5–1 mm	-	118/118 (100%)	109/118 (92.37%)	7/118 (5.93%) Severe vaginal bleeding (n = 7)	NR	1.33 (1.17–1.61)
Sun, 2023 [43]	China	Prospective cohort study	22	33.72 (3.94)	48.00 (34–79)	GS	particles 1–1.4 mm	-	22/22 (100%)	22/22 (100%)	1/22 (4.54%) Severe pain (n = 1)	6/22 (27.27%)	NR
Rahman, 2023 [44]	China	Retrospective cohort study	137	30.3 (0.72)	NR	GS	particles	-	137/137 (100%)	127/137 (92.7%)	NR	82/137 (59.85%)	1.39 (1.02)
Hong, 2022 [45]	China	Retrospective cohort study	160	33.1 (5.1)	51.1 (12.3)	GS	particles 1–2 mm	-	160/160 (100%)	158/160 (98.75%)	NR	NR	1.43 (0.43)
Gu, 2022 [46]	China	Retrospective cohort study	54	31.4 (3.9)	51.91 (21.78)	GS	particles 0.56–0.71 mm	-	54/54 (100%)	54/54 (100%)	NR	32/54 (59.30%)	1.12 (0.29)
Zhou, 2022 [47]	China	Retrospective cohort study	85	32.7 (5.4)	53.2 (13.8)	GS	particles 1–2 mm	-	85/85 (100%)	75/85 (88.23%)	7/85 (8.23%) Severe vaginal bleeding (n = 6) Leg embolization (n = 1)	NR	NR
Shao, 2022 [48]	China	Retrospective cohort study	101	33.5 (9.2)	55.2 (15.9)	GS	particles	-	101/101 (100%)	95/101 (94.06%)	0/101 (0%)	19/101 (18.9%)	NR
Wang, 2021 [49]	China	Retrospective cohort study	23	29.2 (3.60)	NR	GS	NR	-	23/23 (100%)	21/23 (91.3%)	2/23 (8.7%) Massive hemorrhage (n = 2)	NR	NR
Yin, 2020 [50]	China	Retrospective cohort study	42	NR	NR	GS	particles and strips	-	42/42 (100%)	40/42 (95.24%)	NR	NR	NR
Fang, 2020 [51]	China	Case series	32	30.39 (5.78)	68.05 (23.29)	GS	particles	-	32/32 (100%)	14/32 (43.75%)	5/32 (15.62%) Massive hemorrhage (n = 5)	NR	NR
Li, 2020 [52]	China	Retrospective cohort study	169	33.58(4.88)	NR	GS	particles 0.56–0.71 mm and 1 mm	-	169/169 (100%)	162/169 (96%)	5/169 (2.96%) Massive vaginal bleeding (n = 2) Amenorrhea (n = 2) Bacteremia (n = 1)	101/169 (59.70%)	NR
Ou, 2020 [53]	China	Prospective cohort study	65	34 (4.40)	52.29 (10.32)	GS	particles 0.5–1 mm	-	65/65 (100%)	64/65 (98.46%)	0/65 (0%)	NR	NR
Qiu, 2019 [54]	China	Retrospective cohort study	62	32.24 (4.91)	-	GS	particles 0.9–1.2 mm	-	62/62 (100%)	55/62 (88.71%)	4/62 (6.45%) Massive vaginal bleeding (n = 4)	NR	1.17 (0.25)
Xiao, 2019 [55]	China	Retrospective case-control study	35	32.67(6.96)	51.50 (44–62)	GS	particles	-	35/35 (100%)	35/35 (100%)	0/35 (0%)	NR	NR
Zhang, 2019 [56]	China	Retrospective cohort study	46	32.5 (4.70)	48.7 (9.80)	GS	particles	-	46/46 (100%)	46/46 (100%)	0/46 (0%)	NR	NR
Tumenjargal, 2018 [57]	Japan	Retrospective cohort study	33	33 (4.20)	43.90 (8.30)	GS	particles	-	33/33 (100%)	29/33 (87.9%)	0/33 (0%)	NR	1.2 (0.64)
Gao, 2018 [58]	China	Retrospective cohort study	57	33.46(4.47)	54.25 (11.60)	GS	NR	-	57/57 (100%)	57/57 (100%)	0/57 (0%)	NR	NR
Guo, 2018 [5]	China	Retrospective cohort study	51	32.21(5.68)	54.82 (9.27)	GS	particles	-	51/51 (100%)	41/51 (80.4%)	0/51 (0%)	NR	NR
Hong, 2017 [59]	China	Retrospective cohort study	67	31.74(3.69)	NR	GS	particles	-	67/67 (100%)	59/67 (88.06%)	3/67 (4.48%) Severe fever (n = 3)	NR	1.16 (0.20)
Ma, 2017 [33]	China	Retrospective cohort study	22	32 (29–35)	49.00 (42–63)	GS	particles 0.56–0.71 mm	-	22/22 (100%)	19/22 (86.36%)	2/22 (9.09%) Severe vaginal bleeding (n = 2)	2/22 (8.30%)	2 (1.50–2.83)
Chen, 2017 [12]	China	Retrospective cohort study	49	33.7 (4.80)	NR	GS	particles 1 mm	-	49/49 (100%)	47/49 (95.92%)	2/49 (4.08%) Massive hemorrhage (n = 2)	35/49 (71.40%)	NR
Liu, 2016 [60]	China	Retrospective cohort study	38	NR	NR	GS	particles	-	38/38 (100%)	38/38 (100%)	NR	NR	NR
Qi, 2015 [27]	China	Case series	28	31.68 (4.58)	54.33 (17.51)	GS	particles 1–2 mm	-	28/28 (100%)	25/28 (89.3%)	5/28 (17.86%) Massive hemor-rhage (n = 4) Non target embolization (n = 1)	NR	0.67–1.50
Qian, 2015 [61]	China	Prospective clinical study	66	31.39 (4.22)	51.66 (9.35)	GS	particles	-	66/66 (100%)	63/66 (95.45%)	1/66 (1.51%) Hysterectomy due to hemorrhagic shock (n = 1)	NR	NR
Zhu, 2016 [62]	China	Retrospective cohort study	46	31.4 (5.10)	60.6 (16.40)	GS	particles	-	46/46 (100%)	45/46 (97.83%)	2/46 (4.35%) Severe fever (n = 1) Massive vaginal bleeding (n = 1)	NR	1.06 (0.36)
Wang, 2024 [63]	China	Retrospective cohort study	45	31.56 (2.22)	54.25 (15.54)	GS+ MTX	particles	MTX 100 mg ia	45/45 (100%)	45/45 (100%)	0/45 (0%)	3/45 (6.67%)	1.63 (0.16)
Sun, 2023 [43]	China	Prospective cohort study	22	32.67 (4.04)	46 (35–90)	GS + MTX	particles 1–1.4 mm	MTX 1 mg/Kg ia	22/22 (100%)	22/22 (100%)	2/22 (9.09%) Severe pain (n = 2)	9/22 (40.91%)	NR
Baffero, 2023 [64]	Italy	Retrospective cohort study	11	35 (29–38)	45 (41–49)	GS + MTX	particles 0.5–1 mm	MTX 50 mg ia	11/11 (100%)	11/11 (100%)	0/11 (0%)	NR	1.43 (1–1.73)
Tan, 2021 [65]	China	Prospective non-randomized study	36	33.10(3.90)	54.44 (9.50)	GS + MTX	particles 0.56–1.4 mm	MTX 50 mg ia	36/36 (100%)	35/36 (97.22%)	2/36 (5.55%) Severe blood loss (n = 1) Pelvic infection (n = 1)	NR	NR
Cao, 2021 [66]	China	Retrospective cohort study	53	34.79 (3.43)	49.43 (6.38)	GS + MTX	particles 1–1.4 mm	MTX 50 mg ia	53/53 (100%)	52/53 (98.11%)	1/53 (1.89%) Heavy vaginal bleeding (n = 1)	NR	NR
Cheng, 2020 [67]	China	Retrospective cohort study	61	33.50 (0.60)	52 (42–58)	GS + MTX	particles 1 mm	MTX 200 mg ia	61/61 (100%)	50/61 (82%)	3/61 (4.91%) Laparotomy due to hemorrhage or bladder injury (n = 3)	NR	NR
Lou, 2020 [68]	China	Retrospective cohort study	53	33 (3.60)	47 (8.40)	MTX → GS *	particles	MTX 50 mg/m^2^ BSA ia or im	53/53 (100%)	52/53 (98.11%)	3/53 (5.66%) Severe bleeding (n = 2) Massive hemorrhage (n = 1)	NR	1.75 (1.1)
Wang, 2019 [69]	China	Retrospective cohort study	38	31.78 (2.57)	55.04 (10.76)	GS + MTX	particles	MTX 25 mg ia	38/38 (100%)	38/38 (100%)	6/38 (15.79%) DVT (n = 2) Hypo- or a-menorrhea (n = 3) Ovarian failure (n = 1)	NR	NR
Fei, 2019 [70]	China	Retrospective cohort study	26	31.4 (4.40)	NR	GS + MTX	particles	MTX 50 mg ia	26/26 (100%)	26/26 (100%)	0/26 (0%)	NR	NR
Gao, 2018 [58]	China	Retrospective cohort study	36	32.18(5.65)	55.58 (9.82)	GS + MTX	NR	MTX 150 mg ia	36/36 (100%)	36/36 (100%)	0/36 (0%)	NR	NR
Li, 2018 [71]	China	Retrospective cohort study	383	32.3 (4.90)	NR	GS + MTX	particles 0.5–1 mm	MTX 50–70 mg ia	377/383 (98.4%)	379/383 (99%)	16/383 (4.18%) Massive hemorrhage (n = 11) Severe fever (n = 5)	167/383 (43.52%)	NR
Xiao, 2018 [72]	China	Retrospective cohort study	102	33.1 (4.60)	51.19 (11.13)	GS + MTX	particles 0.7–1 mm	MTX 100–150 mg ia	102/102 (100%)	98/102 (96.08%)	4/102 (3.92%) Laparotomy (n = 4)	NR	NR
Xiao, 2017 [73]	China	Retrospective cohort study	45	31.87 (4.50)	48.76 (8.63)	GS + MTX	particles 0.5–1 mm	MTX 50 mg ia	45/45 (100%)	44/45 (97.78%)	6/45 (13.33%) Heavy vaginal bleeding (n = 1) Severe pain (n = 2) Amenorrhea (n = 3)	8/45 (16.67%)	1.40 (0.61)
Yang, 2016 [74]	China	Retrospective cohort study	77	NR	NR	GS + MTX	particles	MTX 50 mg ia	77/77 (100%)	77/77 (100%)	0/77 (0%)	NR	NR
Du, 2015 [75]	China	Retrospective case-control study	175	32.44(4.60)	54.05 (14.04)	GS + MTX	particles 1.4–2 mm	MTX 1 mg/Kg ia	175/175 (100%)	169/175 (96.57%)	6/175 (3.43%) Massive hemorrhage (n = 6)	NR	NR
Huang, 2015 [76]	China	Retrospective cohort study	31	32.42(5.94)	42.12 (6.32)	GS + MTX	particles 0.5–1 mm	MTX 50 mg/m2 BSA ia	31/31 (100%)	31/31 (100%)	0/31 (0%)	NR	NR
Sun, 2015 [77]	China	Retrospective cohort study	15	31.70(1.70)	43.70(1.40)	GS + MTX	particles	MTX 100 mg ia	15/15 (100%)	11/15 (73.33%)	4/15 (26.67%) Severe vaginal bleeding (n = 4)	NR	NR
Wang, 2015 [78]	China	Prospective randomized controlled trial	24	29.96 (4.14)	51.90(2.90)	GS + MTX	particles	MTX 25 mg ia	24/24 (100%)	20/24 (83.33%)	NR	NR	NR
Guo, 2015 [79]	China	Case series	50	NR	56.78 (17.43)	GS ± MTX	particles 1–2 mm	MTX 50 mg ia	50/50 (100%)	42/50 (84%)	5/50 (10%) Severe vaginal bleeding (n = 4) Amenorrhea (n = 1)	NR	NR
Qi, 2015 [27]	China	Case series	22	31.68 (4.58)	59.86 (17.67)	GS + MTX	particles 1–2 mm	MTX 50 mg ia	22/22 (100%)	17/22 (77.3%)	1/22 (4.54%) Hysterotomy (n = 1)	NR	0.67–1.50
Cao, 2018 [80]	China	Retrospective cohort study	101	32.98 (4.96)	NR	GS + PVA	particles	-	101/101 (100%)	99/101 (98.01%)	4/101 (3.96%) Massive hemorrhage (n = 2) Amenorrhea (n = 2)	60/101 (59.40%)	1.48 (0.9)

**Footnotes** -: not applicable; BSA: body surface area; DVT: deep vein thrombosis; GS: gelatin sponge; ia: intra-arterial administration; im: intra-muscular administration; MTX: methotrexate; NR: not reported; PVA: polyvinyl alcohol. * UAE was performed 1 to 16 days after the administration of MTX. Outcome fields are reported as n/N (%) wherever available; otherwise, the cell is labeled “NR”. In many cohorts, GS form/size is described generically as “GS particles” without further details.

## 3. Gelatin Sponge Preparation Methods

### 3.1. Source and Processing Considerations

Gelatin sponge (GS) is an absorbable, water-insoluble embolic agent derived from porcine or bovine collagen [81]. Commercial absorbable gelatin sponge devices used in embolization practice are typically manufactured from purified animal-derived gelatin (most commonly porcine), processed into a porous, compressible matrix and sterilized. For example, Gelfoam^®^ is described as a porous absorbable gelatin sponge prepared from purified porcine skin gelatin, while SPONGOSTAN™ is similarly described as a porcine gelatin absorbable sponge [82,83,84].

Beyond the operator-dependent preparation method (pledgets/slurry/torpedoes), manufacturing variables (e.g., matrix density/porosity, mechanical integrity, and any cross-linking or structural stabilization when present) can theoretically influence (i) swelling and compressibility, (ii) fragmentation during pumping/slurry preparation (thereby affecting the effective particle-size distribution and distal penetration), and (iii) in vivo persistence and recanalization timing. Cross-linking is a well-established determinant of degradation kinetics in gelatin-based embolic materials, with higher cross-linking generally associated with slower degradation and longer embolic persistence; therefore, differences in gelatin processing may contribute to variability in embolization behavior even when “gelatin” is reported as the embolic agent [85].

Importantly, most CSP cohorts do not report the device brand, animal source, or processing details, which prevents direct comparative assessment; we, therefore, encourage standardized reporting of these elements in future studies.

### 3.2. General Preparation Techniques in Embolotherapy

**Figure 1:**
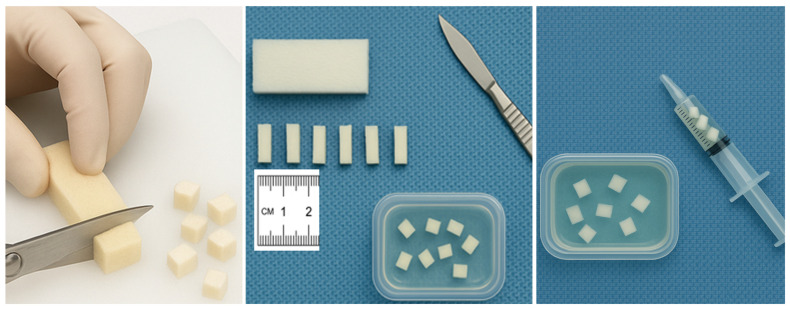
Hand-cut method (pledgets).

GS has been widely utilized in transcatheter arterial embolization (TAE) across various clinical indications—including uterine fibroids, hepatocellular carcinoma, and postpartum hemorrhage—due to its hemostatic properties and biodegradability [17,20,86]. However, its clinical safety and effectiveness are influenced by how it is prepared before administration [87]. In our review, most CSP cohorts (46/50, 92%) reported the use of “GS particles”, while detailed preparation steps (pre-sized vs. custom-made; cut size, pumping steps, solvent, and injection pressure) were frequently underreported. Among multiple preparation techniques described in the literature and clinical practice for embolotherapy, the following three are most commonly utilized:

- Hand-Cut Method (Pledgets):

This technique involves manually slicing GS sheets into uniform 1–2 mm cuboidal fragments using scissors or a scalpel. These pledgets are suspended in contrast, drawn into a syringe, and injected through a catheter (Figure 1). This method provides a relatively predictable particle size. This reduces the risk of unintended distal embolization and promotes safer embolization in uterine interventions, thereby minimizing the likelihood of excessive tissue ischemia. A retrospective study by Saiga et al. (2018) demonstrated zero incidence of intrauterine synechiae in UAE using pledgets versus 83.3% in slurry-prepared GS, underlining its importance in fertility preservation [88].

**Figure 2:**
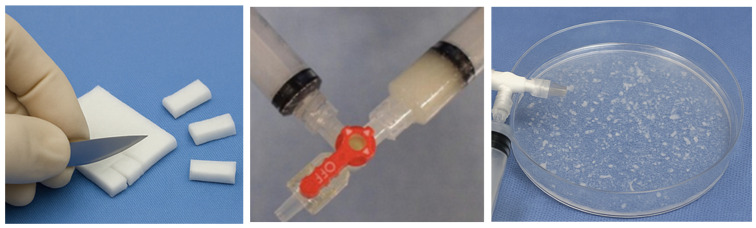
Pumping method (slurry).

- Pumping Method (Slurry):

This widely used method involves fragmenting GS sheets into irregular pieces or using pre-shaped small cubes or pieces made of GS, followed by emulsification by repetitive pumping between syringes containing contrast medium and connected via a three-way stopcock (Figure 2). This creates a heterogeneous slurry of particles—ranging from <100 µm to >1000 µm—with a high proportion of fine fragments. Although this method is fast and suitable in urgent settings, the small and inconsistent particle sizes increase the risk of distal embolization. This potentially affects endometrial or ovarian blood supply. Interestingly, Miyayama et al. (2014) and Saiga et al. (2018) reported that slurry may result in greater ischemic damage due to distal vessel penetration, increasing risk of intrauterine adhesions, necrosis, and compromised reproductive function [88,89].

**Figure 3:**
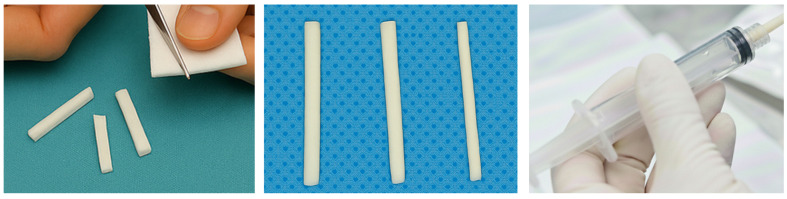
Torpedo method.

- Torpedo Method:

This preparation involves using pre-shaped or custom-made torpedoes by shaping the GS sheet into cylindrical torpedoes that match the internal diameter of the delivery catheter. These torpedoes are gently pushed with contrast to lodge within targeted arteries (Figure 3) [90]. The method provides proximal embolizationbut less risk of catheter occlusion than large pledgets. Torpedoes provide an intermediate embolic profile—larger and more uniform than slurry particles, but less bulky than hand-cut pledgets—making them suitable when a balance between penetration, speed and safety is desired. However, literature is lacking on specific reproductive outcomes in CSP regarding this preparation technique [91].

Furthermore, commercial GS embolic products are available as pre-sized particles, typically in spherical or cuboidal forms, ranging from 0.5 to 2 mm. These products offer a standardized alternative to manual preparation, ensuring uniform particle dimensions, reduced preparation time, and greater reproducibility. While mainly approved for certain indications like hepatic embolization in some regions, they are often used off-label for uterine artery embolization (UAE) in CSP and fibroid treatment [92]. The use of pre-sized products may help mitigate the risks associated with operator-dependent variability and particle fragmentation, although long-term fertility outcomes remain under investigation. In their retrospective cohort study evaluating UAE with pre-sized GS particles of 1–2 mm on 160 patients with CSP, Hong et al. observed an average of 1.43 months for the menstrual cycle to resume [45]. GS can be blended with iodized contrast, saline, or a mixture of the two. This allows for the modification of its concentration in the chosen liquid while making the solution radiopaque [89].

### 3.3. Clinical Implications in Uterine Artery Embolization for Cesarean Scar Pregnancy

The method of GS preparation has significant implications in UAE, especially for fertility-preserving indications such as cesarean scar pregnancy (CSP). Studies indicate that particle size and uniformity influence not only embolization efficacy but also post-procedural complications. A pivotal study by Saiga et al. (2019) compared slurry and GS pledgets in UAE for postpartum hemorrhage [88]. They found a strikingly higher incidence of intrauterine synechia (83.3%) in the slurry group versus 0% in the pledget group, suggesting that small slurry particles may occlude microvasculature and compromise endometrial integrity [88]. This concern extends to CSP, where preservation of fertility is paramount. Small GS particles may inadvertently embolize collateral branches, including the utero-ovarian and cervical arteries. This can potentially lead to ovarian failure, uterine necrosis, or adhesions [93]. In contrast, larger pledgets or torpedoes are less likely to migrate beyond intended vascular territories. Mathieu et al. (2022) demonstrated that proximal embolization using GS torpedoes in retained products of conception achieved 100% clinical success with minimal complications, supporting their use in uterine conditions where perfusion preservation is desirable [91]. Moreover, Miyayama et al. (2014) recommend using GS particles ≥0.5 mm to reduce ischemic complications in TAE, highlighting that permanent occlusion may still occur depending on inflammation and vessel remodeling—even with GS intended as a temporary agent [89]. Historical data also support the safety of pledget use in obstetric embolotherapy: Stancato-Pasik et al. (1997) reported resumed menses in 92% and successful term pregnancies in all women attempting conception after embolization using pledgets [94].

**Figure 4:**
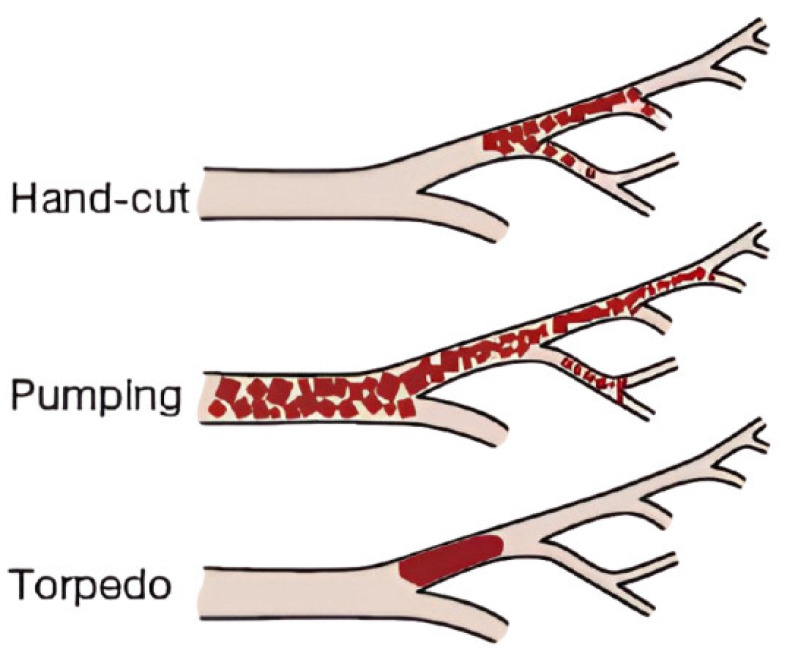
Schematic representation of embolization distribution patterns based on gelatin sponge preparation techniques.

Table 2 summarizes the advantages, disadvantages, and clinical notes associated with three primary techniques of GS preparation: hand-cut (pledgets), pumping method (slurry), and torpedoes. These methods differ in particle size control, embolization depth, preparation complexity, and safety profile, especially in fertility-preserving procedures like uterine artery embolization (UAE) for cesarean scar pregnancy (CSP). Selection of the appropriate method should be guided by the clinical context, target vascular anatomy, and risk of non-target embolization. Furthermore, Figure 4 illustrates the vascular distribution of embolic material in TAE when using three different GS preparation methods. Hand-cut pledgets tend to achieve proximal-to-distal embolization, occluding both large- and medium-caliber branches. The pumping method (slurry) disperses small particles distally, reaching the microvasculature and potentially causing diffuse occlusion. The torpedo technique results in localized proximal occlusion, with minimal distal penetration, suited for rapid hemostasis and preservation of downstream perfusion. These patterns reflect the differing hemodynamic impacts and clinical utility of each technique, especially in UAE for conditions such as CSP.

Hence, GS preparation significantly impacts the safety and efficacy outcomes of UAE, especially in fertility-sensitive scenarios like CSP. Pledgets and torpedoes are preferred over slurry when aiming to minimize ischemic complications such as intrauterine synechia or ovarian failure, unless rapid hemostasis is needed and fertility preservation is not a concern. Commercially pre-sized GS products may standardize practice. Establishing procedural standards for GS preparation in CSP can help optimize patient outcomes while preserving reproductive potential.

## 4. Technical Aspects of Uterine Artery Embolization (UAE)

UAE represents a cornerstone in the conservative endovascular management of CSP. While the efficacy and safety of GS as an embolic agent have been widely discussed, a detailed understanding of the procedural technicalities is essential for optimizing patient outcomes, minimizing complications, and ensuring fertility preservation. This section delves into the state-of-the-art procedural framework, encompassing pre-interventional, intra-procedural, and post-procedural management, with a focus on technical execution.

Prior to the procedure, a thorough multidisciplinary evaluation is vital. This involves detailed obstetric and gynecologic history-taking, gynecological examination, and high-resolution imaging studies. Magnetic resonance imaging (MRI) with gadolinium contrast is the preferred modality for visualizing myometrial integrity, delineating the vascular supply, and detecting utero-ovarian or other anastomoses, which may interfere with the completeness of embolization [95,96]. Transvaginal ultrasonography remains useful for immediate diagnosis and anatomical guidance, as it is readily accessible at many facilities. However, it is less effective for procedural planning in complex cases and is limited by interobserver variability [97,98,99,100].

Prophylactic antibiotic administration is routinely adopted. A standard regimen includes intravenous cefazolin 1 g administered 30 to 60 min prior to the procedure, though clindamycin is used in penicillin-allergic individuals. In the context of CSP, where infection risk is elevated due to potential extensive necrosis, this prophylaxis becomes critical. Each patient should undergo urinary catheterization to mitigate discomfort, prevent bladder distension with contrast medium from obscuring the uterus, and minimize the required radiation dose [96]. The procedure is typically conducted in a digital subtraction angiography (DSA) suite using fluoroscopic guidance. Conscious sedation is generally adequate, using short-acting benzodiazepines and opioids, such as midazolam and fentanyl. Neuraxial anesthesia (spinal) with an indwelling epidural catheter for post-procedural analgesia is often performed. However, general anesthesia may be indicated in highly anxious patients or prolonged interventions. Providers may choose to premedicate to address symptoms like nausea, potentially using a preventative 4 mg intravenous dose of ondansetron [95,98].

Access is most commonly achieved via the common femoral artery using a 5–6 French vascular sheath, although transradial access (TRA) has gained attention due to lower complication rates and early ambulation benefits. Following vascular access, an aortogram is often performed to visualize the aortoiliac bifurcation and identify vascular anomalies. Selective catheterization of the internal iliac artery is performed with a 4–5 French Cobra, Roberts uterine, or another diagnostic catheter based on operator preference, followed by superselective catheterization of the uterine artery with a 2.4–2.8 French microcatheter system (e.g., Progreat or Renegade HI-FLO) [96,101]. A critical aspect of UAE is understanding uterine artery anatomy, which typically arises as the first or second branch of the anterior division of the internal iliac artery and involves a U-shaped course with a descending, transverse, and ascending segment. The uterine artery often gives off the cervicovaginal branch (arises from the transverse segment) and the utero-ovarian anastomoses. Protective coiling or use of >700 μm particles may reduce the risk of unintended damage to the ovaries [95,102,103,104,105]. After confirming microcatheter placement via contrast injection, embolization is carried out using the embolic agent, delivered under fluoroscopic control. The goal is to achieve a “pruned tree” appearance indicating distal branch occlusion with sluggish antegrade flow but avoiding complete stasis, which may increase the risk of non-target embolization and ischemia to adjacent structures [102,106]. Completion angiography is crucial to confirm technical success, defined as cessation or marked reduction in flow in both uterine arteries. It is worth noting that bilateral UAE is advised, unless clear unilateral feeding to CSP is observed. In some cases, repeat UAE or embolization of collateral vessels supplying gestational sac may be necessary to achieve complete devascularization, although bilateral ovarian embolization is avoided to preserve ovarian function. Non-gonadal collaterals supplying gestational sac may include vesical, internal pudendal, and lumbar arteries [95,96,107].

Post-procedural management includes monitoring for pain, fever, and signs of infection. Analgesia is managed with NSAIDs and opioids as needed. Postembolization syndrome—characterized by pelvic pain, low-grade fever, malaise, and leukocytosis—is common but self-limiting. Follow-up imaging (typically MRI or ultrasound) is advised within 4–6 weeks, regardless of CSP-related diagnostic imaging, to assess uterine involution and exclude complications such as retained products of conception, infectious disease (endometritis, pelvic inflammatory disease, tubo-ovarian abscess, and pyomyoma), ovarian dysfunction, and uterine necrosis [108,109].

In conclusion, the technical nuances of UAE are critical in managing CSP safely and effectively, since the procedure remains highly operator-dependent, and familiarity with pelvic vascular anatomy and embolization techniques is essential to optimize results while minimizing risk.

## 5. Efficacy Outcomes

Across the published series, authors describe the following three recurring embolization strategies: UAE with GS alone, GS combined with MTX, and GS combined with a permanent embolic such as PVA. Given the heterogeneity of study designs, patient selection, adjunct treatments, and follow-up, we did not perform a formal meta-analysis. Instead, outcomes were synthesized descriptively as reported, and stratified by embolization strategy to improve interpretability. Overall, 50 studies involving 3139 patients were included in the review. Quantitative analysis demonstrated an overall pooled technical success rate of 99.8% (95% CI: 99.7–99.9%) and a pooled clinical success rate of 94.8% (95% CI: 94.0–95.6%). Across the studies in Table 1, UAE achieved near-universal technical success. This was reflected in the pooled rates for both the GS group (n = 1733) and the GS + PVA group (n = 101), which reached 100%. One exception was a large GS + MTX series with a 98.4% success rate [71]. This result slightly adjusted the pooled technical success for the GS + MTX group (n = 1305) to 99.5% (95% CI: 99.1–99.9%). Clinical success—defined as control of CSP without massive hemorrhage, hysterectomy, major surgery, or unplanned treatments—was also consistently high, though stratified analysis revealed slight variations: 93.5% (95% CI: 92.3–94.7%) for GS alone, 96.2% (95% CI: 95.2–97.2%) for GS + MTX, and 98.0% (95% CI: 95.3–100%) for GS + PVA. While results were frequently at or near 100% in both small and large cohorts, center- and design-dependent variability exists. It is plausible that heterogeneity in GS preparation (e.g., particle size 0.5–2 mm, occasional use of strips), differences in gestational age at treatment, and the inherent limitations of retrospective reporting (selection, non-uniform outcome definitions, variable follow-up) contribute to this dispersion in clinical outcomes. To address this variability across study designs and patient populations, we stratified our analysis by treatment strategy (GS alone vs. GS combined with MTX or PVA) and prioritized granular data extraction where available.

When GS is used alone, technical success was uniformly 100% across all reporting studies. Clinical control is often complete, including several series with 100% success (e.g., Pecorino 2024 [38]; Rui 2024 [40]; Sun 2023 [43]; Xiao 2019 [55]; Zhang 2019 [56]; Gao 2018 [58]), and remains high in larger retrospective cohorts such as Hong 2022 (98.75%, n = 160) [45] and Li 2020 (96%, n = 169) [52]. Moderate estimates are observed in some experiences (e.g., Wang 2023 [42]; Zhou 2022 [47]; Hong 2017 [59]; Ma 2017 [33]; Tumenjargal 2018 [57]; Guo 2018 [5]), and a clear outlier (Fang 2020 [51]) reports 43.75% despite 100% technical success. While direct between-study comparisons are constrained by retrospective design, it is reasonable to speculate that variations in GS particle size and form (including studies listing “particles and strips” [50]) and treatment at higher gestational ages (e.g., Fang 2020 [51] reports a mean ~68 days) may have increased procedural difficulty and reduced the probability of single-session clinical resolution. Of note, prospective evidence within this strategy aligns with high effectiveness: Sun 2023 (n = 22) reports 100% technical and clinical success [43]; Ou 2020 (n = 65) 100% technical and 98.46% clinical [53]; and Qian 2015 (n = 66) 100% technical and 95.45% clinical [61]. These prospective datasets, though smaller than the largest retrospectives, help mitigate concerns that high clinical success with GS alone reflects selection bias. Western data (Pecorino 2024, Italy) corroborated feasibility and effectiveness with 100% technical and clinical success [38].

In studies combining GS with methotrexate, technical success was predominantly 100%, with Li 2018 (n = 383) at 98.4% [71]. Clinical success is very high across most series—commonly 100% (Wang 2024 [63]; Sun 2023 [43]; Baffero 2023 [64]; Gao 2018 [58]; Fei 2019 [70]; Yang 2016 [74]) or in the ~96–99% range for several large cohorts (Xiao 2018 [72]; Cao 2021 [66]; Lou 2020 [68]; Du 2015 [75]). A wider spread emerges in a minority of reports, including Cheng 2020 (82%) [67], the randomized trial by Wang 2015 (83.33%) [78], and Sun 2015 (73.33%) [77]. Such dispersion likely reflects protocol heterogeneity (e.g., MTX dose from 25 mg to 200 mg or weight-based regimens; intra-arterial vs. intramuscular administration), non-standardized GS particle sizes (0.5–2 mm with variable cut-offs), and differences in case mix, including gestational age. The retrospective nature of most series, occasional mixed designs (e.g., “± MTX” [67]), and center-specific definitions of clinical success further complicate interpretation. Nonetheless, prospective data within this group support overall efficacy: Tan 2022 (non-randomized; 97.22%) [65] and Sun 2023 (100%) [43] demonstrate high clinical control, while the RCT by Wang 2015 (83.33%) [78] cautions that operational choices (timing, dosing, and patient selection) can materially affect outcomes.

Evidence for GS combined with PVA is limited to a single cohort in Table 1. Cao 2018 (n = 101) reports 100% technical success and 98.01% clinical success [80], indicating excellent effectiveness in that series. While encouraging, broader inferences for this combination are constrained by the narrow evidence base and the same potential modifiers seen across groups (GS preparation, gestational age, and retrospective reporting).

Taken together, the available series portray GS-based UAE as highly reliable, with very high technical success (~100%) and strong clinical control across study designs and settings. Overall, the aggregate evidence supports the conclusion that GS is a highly effective embolic agent for endovascular management of CSP, regardless of adjunctive agents (i.e., MTX or PVA). While GS is the focus of this review, it is important to contextualize its use alongside permanent embolic agents such as PVA particles or calibrated microspheres (e.g., Embozene). Permanent agents offer highly predictable occlusion levels due to standardized sizing and morphology, which may reduce operator-dependent variability compared to GS [32]. However, the temporary nature of GS-induced occlusion remains a theoretical advantage for fertility preservation by allowing earlier restoration of physiological uterine blood flow [110]. The choice between these materials often reflects a balance between the precision of permanent microspheres and the cost-effectiveness and resorbability of gelatin sponge.

Geographical and practice-pattern considerations are important when interpreting these results. Most included cohorts originated from a single region (predominantly Chinese centers), where patient selection, device availability, adjunctive protocols (e.g., MTX use), and peri-procedural pathways may differ from other healthcare systems. Therefore, while overall efficacy appears consistently high, generalizability to other settings should be interpreted cautiously.

## 6. Safety Outcomes

While UAE with GS is highly effective in managing CSP, safety outcomes remain a pivotal consideration, particularly for patients seeking uterine and fertility preservation. This section outlines the safety profile of GS-based UAE, detailing common adverse events, menstrual and reproductive outcomes, and rare but serious complications.

Post-embolization syndrome is the most commonly observed event, characterized by pelvic pain, low-grade fever, nausea, and general malaise. These symptoms typically present within the first 48 h after the procedure and are self-limiting, resolving with analgesics and supportive care. Studies reported an incidence ranging from 0% to 10%, depending on the embolic load and patient-specific vascular sensitivity [43,59]. Although not directly evaluated, embolization distribution patterns based on GS preparation techniques may play a role in determining post-embolization syndrome. This is because the latter is directly linked to necrosis-induced inflammation [111].

Menstrual cycle disturbances are also reported, though typically transient. Oligomenorrhea may occur in up to 60–70% of cases [12,44]. Amenorrhea was recorded in 11 cases out of 3139 embolizations (namely, 0.35%) [52,69,73,79,80]. Interestingly, menstrual resumption is a key indicator of preserved ovarian and uterine function. Reported restoration of menstruation occurred in most women within 1–2 months [33,63,80], though heterogeneity and recall bias limit conclusions. Notably, RMBV definitions were inconsistent among studies and often not reported.

In terms of reproductive safety, several observational and retrospective studies document subsequent successful pregnancies following UAE for CSP. Interestingly, in their retrospective investigation of 91 CSP patients comparing UAE alone vs. UAE combined with D&C, Gao et al. showed decreased MBV and longer menstrual recovery time in the UAE combined with D&C group, but there was no statistically significant difference in fertility outcomes between the two groups, which suggests a reversible impact on reproductive function [14]. Although fertility-related outcomes are multifactorial and influenced by the underlying uterine integrity and prior obstetric history, GS-based UAE is generally associated with favorable reproductive profiles when performed correctly and without complications, as noted by Chen et al. [13]. Preservation of utero-ovarian anastomoses is pivotal to minimize ischemic-related complications to the ovaries. Among all reviewed investigations, Wang et al. reported only one case of ovarian failure [69]. No case of intrauterine synechia was recorded. However, pregnancy and live-birth outcomes are frequently under-reported and their quantification goes beyond the scope of our review.

Serious complications are rare but clinically significant, generally observed in up to 10% of cases across reports. Uterine necrosis, pelvic abscesses, or inadvertent embolization of non-target vessels such as the bladder or ovaries have been reported in isolated cases [27,52,65,67]. These outcomes are typically associated with aggressive embolization strategies or unrecognized vascular variants. Incidence rates for such severe events remain below 2–4% in most published series. Interestingly, in a multicenter prospective trial by Tan et al. [65], only one case of pelvic infection was recorded. Management, when reported, consisted of supportive measures and targeted therapy (e.g., antibiotics ± drainage for infectious complications) or hysterectomy [93]. Hemorrhage is another relevant concern, particularly when severe bleeding leads to hysterectomy thus determining the clinical failure of UAE, as in the instance reported by Qian et al. [61]. Careful recognition of anastomoses that may interfere with the completeness of embolization is crucial to mitigate this risk. Non-gonadal collaterals supplying the gestational sac may include vesical, internal pudendal, and lumbar arteries [95,96,107]. Completion aortoiliac angiography is mandatory to rule out previously unrecognized collaterals.

Taken together, severe complications after UAE with GS are uncommon, while menstrual function typically returns within a short interval. Most cohorts report severe-complication rates in the 2–4% range, with sporadic higher values in a few series. When reported, reduced menstrual blood volume (RMBV) varies widely across studies. Menstrual recovery is generally prompt, with central estimates most often around 1.1–1.5 months. The constraints of non-uniform outcome ascertainment in largely retrospective datasets may explain the observed variation. The variation in complication rates across the analyzed series may be also attributed to the heterogeneity in GS preparation. Manual methods, such as the “slurry” technique, often produce a wide range of particle sizes, including very fine fragments (<500 µm) that can penetrate deep into the microvasculature. Such distal embolization increases the risk of non-target effects, including endometrial synechiae or ovarian dysfunction [88,89]. Conversely, larger hand-cut pledgets or “torpedoes” tend to provide more proximal occlusion [91], potentially offering a safer profile for patients where maintaining uterine integrity is the priority. Importantly, in CSP cohorts, preparation details are frequently underreported (often described generically as “GS particles”), which limits the ability to perform robust outcome comparisons across preparation techniques.

Quantitative analysis was performed on a pooled population of 2684 patients. From the total cohort of 3139 patients included in the review, 455 patients across six studies were excluded from this specific calculation due to the non-reporting of complication events. In the evaluated cohort, severe complications occurred in 4.1% (95% CI: 3.4–4.9%) of cases. In the subgroup analysis, the GS group (analyzed n = 1302) exhibited a severe complication rate of 3.7% (95% CI: 2.7–4.7%). The GS + MTX group (analyzed n = 1281) showed a slightly higher severe complication rate of 4.6% (95% CI: 3.5–5.7%), while the GS + PVA group (n = 101) reported a rate of 4.0% (95% CI: 0.2–7.8%). With GS used alone, multiple cohorts report no severe complications, and large series generally remain within low single-digit percentages. Examples include 0% in several reports (e.g., Shao 2022 [48]; Xiao 2019 [55]; Zhang 2019 [56]), ~3% in Gao 2023 [41], ~4–6% in Hong 2017 [59], and ~6% in Wang 2023 [42], with higher figures in select series (e.g., 8–9% in Zhou 2022 [47] and Ma 2017 [33], 15.6% in Fang 2020 [51]). RMBV, where captured, spans from ~18–27% [43,48] to ~59–71% in some large Chinese cohorts [12,46,52]. Menstrual recovery consistently clusters around ~1.1–1.4 months [41,42,45,46,54,62], with occasional longer intervals around ~2 months (Ma 2017, 2.0 [1.50–2.83] months [33]). When comparing complication rates between GS alone and GS combined with MTX, the data from Table 1 suggest that the overall safety profile remains comparable across both modalities. In studies combining GS with methotrexate (MTX), severe-complication rates are, likewise, low in most reports but with a broader spread in a minority of cohorts. Recent and large series report 0–5% [58,63,64,70], whereas higher estimates appear in specific experiences (e.g., 15.8% in Wang 2019 [69] and 26.7% in Sun 2015 [77]). RMBV again spans a wide interval—6.7% in Wang 2024 [63], 16.7% in Xiao 2016 [73], and ~43.5% in Li 2018 [71]—consistent with heterogeneous definitions and reporting practices. Menstrual recovery remains favorable and generally parallels GS alone, with central values typically ~1.4–1.75 months [63,68,73] and 1.43 [1–1.73] months in the Italian cohort by Baffero 2023 [64]. Prospective evidence in this group (Sun 2023 [43]; Tan 2022 [65]) and a small RCT (Wang 2015 [78]) support overall safety but also illustrate how protocol choices (MTX dose and route, GS particle sizing, and timing relative to embolization) can shift both complication and RMBV rates. For GS combined with PVA, evidence is limited to a single cohort: Cao 2018 reported 3.96% severe complications, ~59.4% RMBV, and menstrual recovery of 1.48 [0.9] months [80]. While these outcomes align with effective hemostasis and timely return of menses, the solitary dataset and potential confounding from center-specific technique preclude broad generalization.

Overall, the published experience suggests that GS-based UAE for CSP is generally well tolerated, with most adverse events being mild and self-limiting. The procedure offers a high degree of uterine preservation, minimal impact on menstrual regularity, and a low incidence of major complications. Prospective studies reinforce the low risk of serious morbidity and high likelihood of hormonal and anatomic recovery. The differences observed between studies likely reflect several factors. These include procedural practices, such as the method of GS preparation and delivery, and the timing of the intervention during gestation. Furthermore, the interpretive limits of retrospective series—specifically regarding selection bias, non-uniform outcome definitions, and variable follow-up—must be considered. Overall, these data support GS-mediated embolization as a safe and dependable therapeutic approach for CSP, complementing the high efficacy outlined above.

## 7. Limitations and Future Perspectives

Key limitations include the following: (i) the predominance of retrospective designs, with variable follow-up and non-uniform definitions of clinical success and reproductive outcomes; (ii) substantial heterogeneity and underreporting in GS preparation and delivery (particle size, preparation method, injection medium/pressure), which limits cross-study comparability and may contribute to variability in efficacy and complication estimates; (iii) variable outcome definitions and selective outcome reporting across studies, with potential publication bias; (iv) restriction to English-language publications, introducing language bias; and (v) geographical concentration of the evidence base, with most cohorts originating from Chinese centers (47 of 50 included studies: 3085/3139 patients), which may limit generalizability to other healthcare systems (case mix, devices, operator experience, and peri-procedural protocols).

Prospective registries or multicenter RCTs would help validate findings arising from our review. Future studies should prioritize prospective, comparative designs with (i) standardized GS preparation protocols (pledgets/torpedoes vs. slurry; pre-sized particles; and particle size thresholds), (ii) predefined procedural endpoints and collateral management strategies, and (iii) a core outcome set capturing technical/clinical success, graded complications, patient-reported outcomes, time to menstrual recovery, and reproductive endpoints at 12–24 months. Multicenter registries and pragmatic trials would allow a direct comparison between GS and other embolics such as PVA, alongside evaluations of cost-effectiveness, learning curves, radiation-dose optimization, and access routes. Establishing consensus standards for GS preparation and reporting is essential. Such standards will minimize variability and ensure the collection of robust research data in the future. To improve the quality and comparability of future research, we propose a minimal reporting checklist to collect data on the use of GS in UAE, as follows:

- CSP type/classification, gestational age, and baseline β-hCG;
- Embolization strategy (bilateral vs. unilateral; target level);
- Catheter/microcatheter and endpoint definition (e.g., near stasis vs. complete stasis);
- GS brand (if stated), preparation method, intended particle/cube size, solvent (contrast/saline), and injection technique/pressure;
- Adjuncts: MTX (route and dose), other embolics (type and size);
- D&C timing;
- Definitions of technical/clinical success and the assessment window;
- Complications with standardized grading (SIR);
- Follow-up duration, menstrual outcomes definitions, and subsequent pregnancy/live-birth outcomes (if collected).

## 8. Conclusions

UAE with gelatin sponge appears effective and generally safe for the management of CSP when performed by experienced operators, supporting uterus preservation. Clinically, careful attention to embolization endpoints and avoidance of unintended distal embolization are central in fertility-sparing settings. However, standardized GS preparation/reporting and prospective multicenter registries or comparative studies are needed to define best practices and long-term reproductive outcomes more reliably.

## Figures and Tables

**Table 2:** Comparison of gelatin sponge (GS) preparation methods used in transcatheter arterial embolization (TAE).

Feature	Pumping (Slurry)	Hand-Cut (Pledgets)	Torpedoes
Preparation Time	Rapid	Time-consuming	Time-consuming (if custom-made)
Particle Size	Variable (often <0.5 mm)	Uniform (0.5–1–2 mm cubes)	Uniform (delivery catheter caliber)
Risk of Distal Embolization	High	Low	Low
Fertility Risk (UAE Context)	Higher (synechia, necrosis)	Lower	Lower
Reproducibility	Low	Moderate	High
Advantages	Produces smaller particles for distal vessel penetration. Easier and quicker preparation.	Larger particle size ensures proximal vessel occlusion. Reduced risk of non-target embolization.	Uniform shape allows controlled embolization. Reduced catheter clogging risk.
Disadvantages	Less control over particle size distribution. Higher risk of non-target embolization. Shorter occlusion duration.	Labor-intensive and time-consuming. Inconsistent particle sizes. Potential for catheter clogging.	Requires specific tools for shaping. Less effective for distal embolization.
Use Case	Emergency, rapid control Caution in elective fertility-sensitive cases, such as UAE.	Elective Best for controlled embolization in fertility-preserving procedures.	Routine embolotherapy (no data on UAE) Good balance of control and ease

## Data Availability

No new data were created or analyzed in this study. Data sharing is not applicable to this article.

## Supplementary Materials

The following supporting information can be downloaded at: https://www.mdpi.com/article/10.3390/gels12010044/s1, File S1: Prisma Flow Diagram; File S2: Full database search strategies; File S3: Design-informed study quality/risk-of-bias appraisal using an adapted Newcastle–Ottawa Scale (NOS) framework [5,12,27,28,29,30,31,32,33,34,35,36,37,38,39,40,41,42,43,44,45,46,47,48,49,50,51,52,53,54,55,56,57,58,59,60,61,62,63,64,65,66,67,68,69,70,71,72,73,74,75,76,77,78,79,80].

## Abbreviations

BSA	Body Surface Area
CSD	Cesarean Scar Defect
CSP	Cesarean Scar Pregnancy
D&C	Dilation and Curettage
DSA	Digital Subtraction Angiography
DVT	Deep Vein Thrombosis
GS	Gelatin Sponge
IQR	Interquartile Range
i.a.	intra-arterial (administration)
i.m.	intramuscular (administration)
MBV	Menstrual Blood Volume
MRI	Magnetic Resonance Imaging
MTX	Methotrexate
NSAIDs	Nonsteroidal Anti-inflammatory Drugs
PVA	Polyvinyl Alcohol
RCT	Randomized Controlled Trial
RMBV	Reduced Menstrual Blood Volume
SD	Standard Deviation
SIR	Society of Interventional Radiology
TAE	Transcatheter Arterial Embolization
TRA	Transradial Access
UAE	Uterine Artery Embolization
UFE	Uterine Fibroid Embolization
