# Supplementary material for: Transcatheter Arterial Embolization (TAE) of Uterine Artery with Gelatin Sponge for Cesarean Scar Pregnancy: A Current State of the Art Review
Source: Gels. 2026 Jan 1;12(1):44. doi: 10.3390/gels12010044 (PMC12841341; doi:10.3390/gels12010044)
Supplement: Supplementary file 1 [file gels-12-00044-s001.zip › gels-3977778-supplementary.docx]

# Supplementary File S1. Prisma Flow Diagram

#
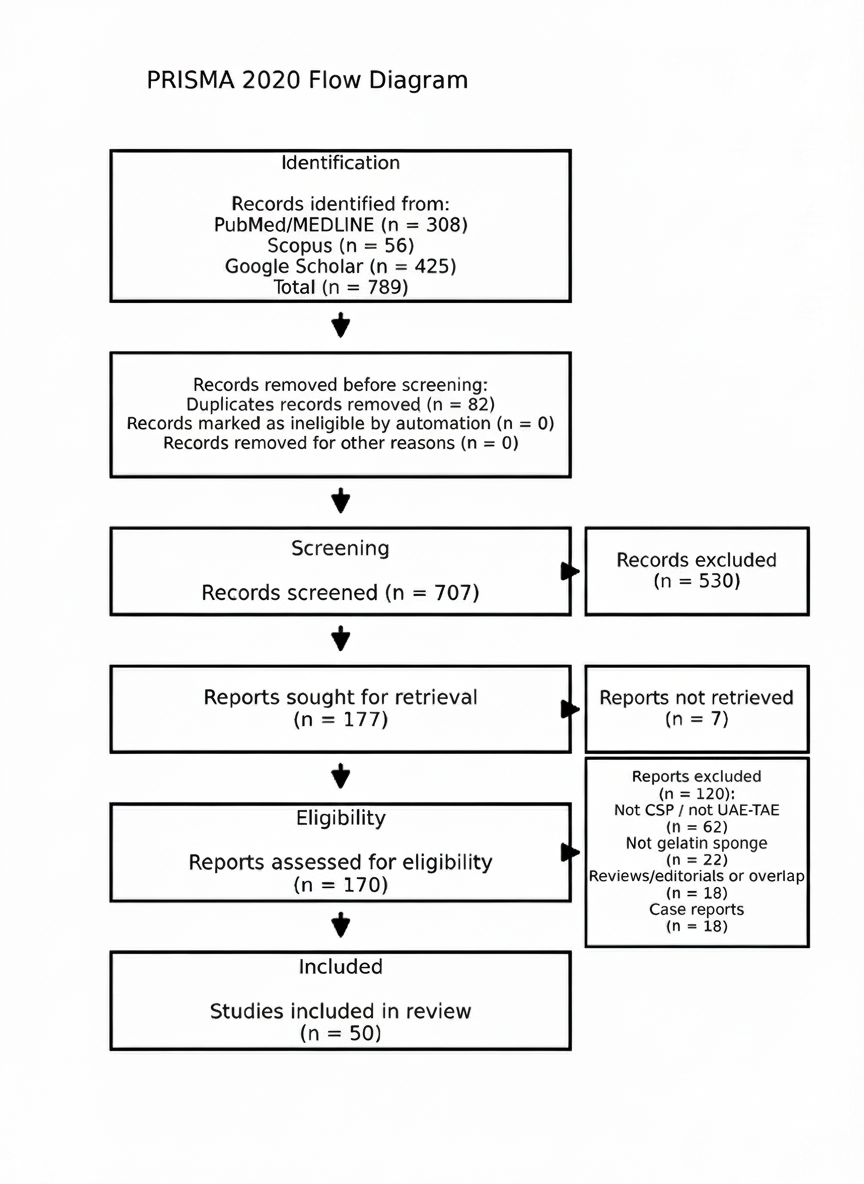


# Supplementary File S2. Full database search strategies

Last search date: 31 December 2024.

## PubMed/MEDLINE

Search string:

("cesarean scar pregnancy" OR "caesarean scar pregnancy" OR "scar pregnancy" OR "ectopic pregnancy" OR "extrauterine pregnancy") AND ("uterine artery embolization" OR "uterine artery embolisation" OR "transcatheter arterial embolization" OR "transcatheter arterial embolisation" OR UAE OR TAE OR embo*)

Filters applied in PubMed interface: Publication date 2015/01/01–2024/12/31; Language: English; Humans.

## Scopus

Search string:

TITLE-ABS-KEY

( ( "cesarean scar pregnancy" OR "caesarean scar pregnancy" OR "scar pregnancy" OR "ectopic pregnancy" OR "extrauterine pregnancy" ) AND ( "uterine artery embolization" OR "uterine artery embolisation" OR "transcatheter arterial embolization" OR "transcatheter arterial embolisation" OR "UAE" OR "TAE" OR "embolization" OR "embolisation" OR "chemoembolisation" OR "chemoembolization" OR "uterine artery" ) AND ( "gelatin sponge" OR "gelfoam" OR "absorbable gelatin sponge" OR "spongostan" OR "absorbable" OR "particles" OR "microspheres" ) )

PUBYEAR > 2014 AND PUBYEAR < 2025

## Google Scholar

Search string :

("cesarean scar pregnancy" OR "caesarean scar pregnancy" OR "scar pregnancy") ("uterine artery embolization" OR "uterine artery embolisation" OR "transcatheter arterial embolization" OR "transcatheter arterial embolisation" OR UAE OR TAE) ("gelatin sponge" OR gelfoam OR "absorbable gelatin sponge")

Titles and abstracts were screened to identify studies published within the time window 2015-2024, relevant to the UAE with GS as a first-line therapy in patients suffering from CSP, while unrelated research was excluded. Keywords such as “gelatin sponge” or “gelfoam” or “absorbable gelatin sponge” were searched to identify the embolic agent used and, where necessary, the full text was also screened.

# Supplementary File S3. Design-informed study quality/risk-of-bias appraisal using an adapted Newcastle–Ottawa Scale (NOS) framework

| **Supplementary Table S1. Study quality / risk-of-bias appraisal** | | | | | |
| --- | --- | --- | --- | --- | --- |
| Approach: design-informed appraisal using an adapted Newcastle–Ottawa Scale (NOS) framework,  given the predominance of observational reports and incomplete reporting of NOS items in the primary literature.  Overall categories reflect study design (RCT=Low; prospective non-randomized=Moderate; retrospective/case series=High). | | | | | |
| Reference | Country | Study design | Sample size (N) | Overall risk of bias  (design-informed; adapted NOS) | Rationale (brief) |
|  |  |  |  |  |  |
| Pecorino, 2024 [38] | Italy | Retrospective cohort Study | 10 | High | Retrospective/case-series design; selection and confounding risks; variable outcome definitions and follow-up; selective reporting possible. |
| Ma, 2024 [39] | China | Retrospective cohort Study | 10 | High | Retrospective/case-series design; selection and confounding risks; variable outcome definitions and follow-up; selective reporting possible. |
| Rui, 2024 [40] | China | Retrospective cohort Study | 39 | High | Retrospective/case-series design; selection and confounding risks; variable outcome definitions and follow-up; selective reporting possible. |
| Gao, 2023 [41] | China | Retrospective cohort Study | 66 | High | Retrospective/case-series design; selection and confounding risks; variable outcome definitions and follow-up; selective reporting possible. |
| Wang, 2023 [42] | China | Retrospective cohort Study | 118 | High | Retrospective/case-series design; selection and confounding risks; variable outcome definitions and follow-up; selective reporting possible. |
| Sun, 2023 [43] | China | Prospective cohort Study | 22 | Moderate | Prospective non-randomized design; potential confounding/selection bias; variable outcome definitions and follow-up. |
| Rahman, 2023 [44] | China | Retrospective cohort Study | 137 | High | Retrospective/case-series design; selection and confounding risks; variable outcome definitions and follow-up; selective reporting possible. |
| Hong, 2022 [45] | China | Retrospective cohort Study | 160 | High | Retrospective/case-series design; selection and confounding risks; variable outcome definitions and follow-up; selective reporting possible. |
| Gu, 2022 [46] | China | Retrospective cohort Study | 54 | High | Retrospective/case-series design; selection and confounding risks; variable outcome definitions and follow-up; selective reporting possible. |
| Zhou, 2022 [47] | China | Retrospective cohort Study | 85 | High | Retrospective/case-series design; selection and confounding risks; variable outcome definitions and follow-up; selective reporting possible. |
| Shao, 2022 [48] | China | Retrospective cohort Study | 101 | High | Retrospective/case-series design; selection and confounding risks; variable outcome definitions and follow-up; selective reporting possible. |
| Wang, 2021 [49] | China | Retrospective cohort Study | 23 | High | Retrospective/case-series design; selection and confounding risks; variable outcome definitions and follow-up; selective reporting possible. |
| Yin, 2020 [50] | China | Retrospective cohort Study | 42 | High | Retrospective/case-series design; selection and confounding risks; variable outcome definitions and follow-up; selective reporting possible. |
| Fang, 2020 [51] | China | Case series | 32 | High | Retrospective/case-series design; selection and confounding risks; variable outcome definitions and follow-up; selective reporting possible. |
| Li, 2020 [52] | China | Retrospective cohort Study | 169 | High | Retrospective/case-series design; selection and confounding risks; variable outcome definitions and follow-up; selective reporting possible. |
| Ou, 2020 [53] | China | Prospective cohort Study | 65 | Moderate | Prospective non-randomized design; potential confounding/selection bias; variable outcome definitions and follow-up. |
| Qiu, 2019 [54] | China | Retrospective cohort Study | 62 | High | Retrospective/case-series design; selection and confounding risks; variable outcome definitions and follow-up; selective reporting possible. |
| Xiao, 2019 [55] | China | Retrospective case-control Study | 35 | High | Retrospective/case-series design; selection and confounding risks; variable outcome definitions and follow-up; selective reporting possible. |
| Zhang, 2019 [56] | China | Retrospective cohort Study | 46 | High | Retrospective/case-series design; selection and confounding risks; variable outcome definitions and follow-up; selective reporting possible. |
| Tumenjargal, 2018 [57] | Japan | Retrospective cohort Study | 33 | High | Retrospective/case-series design; selection and confounding risks; variable outcome definitions and follow-up; selective reporting possible. |
| Gao, 2018 [58] | China | Retrospective cohort Study | 57 | High | Retrospective/case-series design; selection and confounding risks; variable outcome definitions and follow-up; selective reporting possible. |
| Guo, 2018 [59] | China | Retrospective cohort Study | 51 | High | Retrospective/case-series design; selection and confounding risks; variable outcome definitions and follow-up; selective reporting possible. |
| Hong, 2017 [60] | China | Retrospective cohort Study | 67 | High | Retrospective/case-series design; selection and confounding risks; variable outcome definitions and follow-up; selective reporting possible. |
| Ma, 2017 [61] | China | Retrospective cohort Study | 22 | High | Retrospective/case-series design; selection and confounding risks; variable outcome definitions and follow-up; selective reporting possible. |
| Chen, 2017 [62] | China | Retrospective cohort Study | 49 | High | Retrospective/case-series design; selection and confounding risks; variable outcome definitions and follow-up; selective reporting possible. |
| Liu, 2016 [63] | China | Retrospective cohort Study | 38 | High | Retrospective/case-series design; selection and confounding risks; variable outcome definitions and follow-up; selective reporting possible. |
| Qi, 2015 [27] | China | Case series | 28 | High | Retrospective/case-series design; selection and confounding risks; variable outcome definitions and follow-up; selective reporting possible. |
| Qian, 2015 [64] | China | Prospective clinical study | 66 | Moderate | Prospective non-randomized design; potential confounding/selection bias; variable outcome definitions and follow-up. |
| Zhu, 2015 [65] | China | Retrospective cohort Study | 46 | High | Retrospective/case-series design; selection and confounding risks; variable outcome definitions and follow-up; selective reporting possible. |
| Wang, 2024 [66] | China | Retrospective cohort Study | 45 | High | Retrospective/case-series design; selection and confounding risks; variable outcome definitions and follow-up; selective reporting possible. |
| Sun, 2023 [43] | China | Prospective cohort Study | 22 | Moderate | Prospective non-randomized design; potential confounding/selection bias; variable outcome definitions and follow-up. |
| Baffero, 2023 [67] | Italy | Retrospective cohort Study | 11 | High | Retrospective/case-series design; selection and confounding risks; variable outcome definitions and follow-up; selective reporting possible. |
| Tan, 2021 [68] | China | Prospective non-randomized Study | 36 | Moderate | Prospective non-randomized design; potential confounding/selection bias; variable outcome definitions and follow-up. |
| Cao, 2021 [69] | China | Retrospective cohort Study | 53 | High | Retrospective/case-series design; selection and confounding risks; variable outcome definitions and follow-up; selective reporting possible. |
| Cheng, 2020 [70] | China | Retrospective cohort Study | 61 | High | Retrospective/case-series design; selection and confounding risks; variable outcome definitions and follow-up; selective reporting possible. |
| Lou, 2020 [71] | China | Retrospective cohort Study | 53 | High | Retrospective/case-series design; selection and confounding risks; variable outcome definitions and follow-up; selective reporting possible. |
| Wang, 2019 [72] | China | Retrospective cohort Study | 38 | High | Retrospective/case-series design; selection and confounding risks; variable outcome definitions and follow-up; selective reporting possible. |
| Fei, 2019 [73] | China | Retrospective cohort Study | 26 | High | Retrospective/case-series design; selection and confounding risks; variable outcome definitions and follow-up; selective reporting possible. |
| Gao, 2018 [58] | China | Retrospective cohort Study | 36 | High | Retrospective/case-series design; selection and confounding risks; variable outcome definitions and follow-up; selective reporting possible. |
| Li, 2018 [74] | China | Retrospective cohort Study | 383 | High | Retrospective/case-series design; selection and confounding risks; variable outcome definitions and follow-up; selective reporting possible. |
| Xiao, 2018 [75] | China | Retrospective cohort Study | 102 | High | Retrospective/case-series design; selection and confounding risks; variable outcome definitions and follow-up; selective reporting possible. |
| Xiao, 2017 [76] | China | Retrospective cohort Study | 45 | High | Retrospective/case-series design; selection and confounding risks; variable outcome definitions and follow-up; selective reporting possible. |
| Yang, 2016 [77] | China | Retrospective cohort Study | 77 | High | Retrospective/case-series design; selection and confounding risks; variable outcome definitions and follow-up; selective reporting possible. |
| Du, 2015 [78] | China | Retrospective case-control Study | 175 | High | Retrospective/case-series design; selection and confounding risks; variable outcome definitions and follow-up; selective reporting possible. |
| Huang, 2015 [79] | China | Retrospective cohort Study | 31 | High | Retrospective/case-series design; selection and confounding risks; variable outcome definitions and follow-up; selective reporting possible. |
| Sun, 2015 [80] | China | Retrospective cohort Study | 15 | High | Retrospective/case-series design; selection and confounding risks; variable outcome definitions and follow-up; selective reporting possible. |
| Wang, 2015 [81] | China | Prospective randomized controlled Trial | 24 | Low | Prospective randomized design; still limited by small sample size and heterogeneity in adjunct treatments/outcome reporting. |
| Guo, 2015 [82] | China | Case series | 50 | High | Retrospective/case-series design; selection and confounding risks; variable outcome definitions and follow-up; selective reporting possible. |
| Qi, 2015 [27] | China | Case series | 22 | High | Retrospective/case-series design; selection and confounding risks; variable outcome definitions and follow-up; selective reporting possible. |
| Cao, 2018 [83] | China | Retrospective cohort Study | 101 | High | Retrospective/case-series design; selection and confounding risks; variable outcome definitions and follow-up; selective reporting possible. |
